# Antifouling Slippery Surface with Enhanced Stability for Marine Applications

**DOI:** 10.3390/ma17225598

**Published:** 2024-11-15

**Authors:** Yun Li, Yuyang Zhou, Junyi Lin, Hao Liu, Xin Liu

**Affiliations:** State Key Laboratory of High-Performance Precision Manufacturing, Dalian University of Technology, Dalian 116024, China; hebly@mail.dlut.edu.cn (Y.L.); zyuyang12@163.com (Y.Z.); linjy1372@163.com (J.L.); 15141623006@163.com (H.L.)

**Keywords:** slippery coating, nano-silver, antifouling, stability

## Abstract

In recent years, slippery liquid-infused porous surfaces (SLIPSs) have gained significant attention in antifouling applications. However, their slippery performance often deteriorates in dynamic environments, limiting their service life. TC4 titanium alloy, commonly used in hulls and propellers, is prone to biofouling. SLIPSs have gained significant attention in antifouling applications. However, their slippery performance often deteriorates in dynamic environments, limiting their service life. To address these issues, a novel slippery liquid-infused surface (STASL) was developed on TC4 through the integration of hydroxyl end-blocked dimethylsiloxane (OH-PDMS), a silane coupling agent (KH550), and nano-titanium dioxide loaded with silver particles (TiO_2_-Ag, anatase) and silicone oil, thereby ensuring stable performance in both dynamic and static conditions. The as-prepared surfaces exhibited excellent sliding capabilities for water, acidic, alkaline, and saline droplets, achieving speeds of up to 2.859 cm/s. Notably, the STASL demonstrated superior oil retention and slippery stability compared to SLIPS, particularly at increased rotational speeds. With remarkable self-cleaning properties, the STASL significantly reduced the adhesion of proteins (50.0%), bacteria (77.8%), and algae (78.8%) compared to the titanium alloy. With these outstanding properties, the STASL has emerged as a promising solution for mitigating marine biofouling and corrosion on titanium alloys.

## 1. Introduction

Titanium and its alloys, developed in the 1950s, have been lauded for their exceptional strength, low density, corrosion resistance, non-magnetic properties, favorable weldability, sound transmission, and remarkable impact resistance, particularly in marine environments [1]. However, due to the favorable biocompatibility of titanium and its alloys, as well as titanium alloy heat exchangers and condensers that have been in use in marine environments for an extended period, have been observed to develop a layer of marine organic microorganisms on their surfaces. This layer can form a heat insulation layer that exceeds the allowable thermal resistance value at the time of design. Furthermore, the formation of a mucus layer on the pipeline surface not only results in corrosion of the metal but also reduces the pipeline pressure [2]. Consequently, there is a need for special attention to be paid to the biofouling of titanium alloy materials in marine environments. This study focused on TC4 titanium alloy, which is utilized in the fabrication of hulls, propellers, and other related components.

A variety of methods have been proposed to prevent marine biofouling thus far. The principle of anti-fouling technology can be roughly divided into three categories: physical, chemical, and biological anti-fouling methods. Physical anti-fouling methods were employed to diminish or prevent the adhesion of fouling organisms, thereby achieving the goal of anti-fouling. These methods included mechanical removal [3,4], cavitation water jet flow decontamination [5,6], self-stripping coating [7], ultrasonic antifouling [8,9], ultraviolet anti-fouling [10], and electric field antifouling [11]. Physical antifouling methods were associated with several disadvantages, including complex technology, low efficiency, and high cost. Chemical antifouling methods were used to prevent the attachment of marine organisms by selecting specific chemicals capable of poisoning the spores or larvae of fouling organisms. The aforementioned methods could be further classified into the following categories: drug immersion method [12], direct poisoning method [13], electrolytic antifouling method [11,14], and coating protection method [15]. However, these methods were highly toxic to fouling organisms and non-target marine organisms, thereby causing significant damage to marine ecosystems. Biological methods were employed to achieve the objective of preventing biological fouling by inhibiting adhesion, preventing metamorphosis, interfering with nerve conduction, and exerting a repellent action. Research in this field primarily concentrates on the following areas: biological antifouling agents [16,17,18], microstructure antifouling methods [19,20], impermeable antifouling coatings [21], and so forth. The application of biological anti-fouling methods was significantly constrained by their high cost, limited number of documented anti-fouling examples, and the lack of large-scale laboratory experiments conducted on actual ships [22]. It was, therefore, of great significance to develop an efficient and environmentally friendly anti-fouling method for titanium alloys.

Bionic wetting technology is a method of designing surfaces with specific wetting properties, such as superhydrophobic and slippery surfaces, by drawing on the characteristics of animals and plants in nature. Superhydrophobic surfaces have demonstrated significant advancements and applications in various fields, particularly in oil–water separation [23], fluid drag reduction [24,25], and water droplet power generation [26], among others. However, there were some limitations in their resistance to biological fouling. Bers et al. [27] discovered that microorganisms, such as bacteria or diatoms, could gradually penetrate the micro texture structure, thereby destroying its hydrophobic properties. Furthermore, Nguyen et al. [28] discovered that the air entrapped within the surface profile was susceptible to damage under high pressure, resulting in its replacement by water and the subsequent loss of the material’s hydrophobic properties.

Recently, slippery liquid-infused porous surfaces (SLIPSs) were proposed as an alternative to superhydrophobic surfaces [29]. Epstein et al. [30] successfully constructed a SLIPS on a polytetrafluoroethylene (PTFE) film and demonstrated its excellent antibacterial ability. Ouyang et al. [31] successfully prepared a SLIPS on stainless steel substrates. The results of the tests demonstrated that the density of marine bacteria deposited on SLIPS was two orders of magnitude lower than that on bare stainless steel. In a separate study, Li et al. [32] prepared SLIPS with melamine sponge (MS) as the substrate, and the resulting surface demonstrated effective anti-*E. coli* and diatom adhesion properties. Furthermore, slippery surfaces possessed a multitude of other exceptional characteristics. Xiang et al. [33] successfully prepared a solid slippery surface (SSS) by injecting solid lubricant into the micro-nano “Metasequoia” structure. The resulting surface demonstrated excellent corrosion resistance, as evidenced by the test results. Ma et al. [34] put forth the proposition that slippery coatings without loss of fluxes (SCLL) and SCLL evinced resistance to icing and corrosion. Fan et al. [35] prepared WO₃-based slippery coatings via spraying and a photocatalytic reaction. The coating demonstrated remarkable capabilities in water mist capture, water droplet expansion, and the removal of collected water, thereby exhibiting an excellent water collection performance.

The antifouling performance of SLIPS was primarily attributable to the injected lubricant, which was inherently challenging to be tightly combined with the substrate [36]. Furthermore, the shear stress generated by flowing water accelerated the loss of lubricants, thereby reducing the service life of SLIPS. The use of a smooth coating with high substrate adhesion strength represented a potential solution to this problem. However, existing smooth coatings had several shortcomings, including low smoothness, harsh preparation conditions, use of toxic chemical reagents, and unreported resistance to proteins, bacteria, and algae.

This paper introduced a novel approach to antifouling, which was achieved by synergistically combining SLIPS with environmentally friendly antibacterial nano-silver particles. The novel slippery liquid-infused surfaces (STASLs) for antifouling were created using hydroxyl end-blocked dimethylsiloxane (OH-PDMS), a silane coupling agent (KH550), and nano-titanium dioxide loaded with silver particles (TiO_2_-Ag). Anatase TiO_2_ is the more commonly used form, and its (001) crystal surface exhibits greater activity [37]. Considering the limitations of the light source in the marine environment and the excellent antimicrobial properties of silver nanoparticles [38], irrespective of whether they are in a state of illumination, TiO_2_ particles containing nano silver were employed. At this juncture, TiO_2_ serves as the carrier of nano-silver and enhances the mechanical strength of the coating. Rough micron-sized structures were constructed by laser etching and shown to be hydrophobic and lipophilic by testing their contact angles [39]. The rough structures were subsequently filled with a lubricant (silicone oil) to form the STASL and were able to withstand significant mechanical stress. This method was advantageous due to its simplicity, low cost, and environmental friendliness, while the nanoparticles enhanced mechanical strength and biofouling resistance. The STASL exhibited excellent smoothness for various droplets and superior anti-protein, anti-bacterial, and anti-algal adhesion properties. This made them a promising solution for marine antifouling applications on titanium alloys.

## 2. Materials and Methods

### 2.1. Materials

Titanium alloy plates (Ti-6Al-4V#, TC4, 30 × 60 × 2 mm^3^) were purchased from Dongguan Zhuoyang Precision Technology Co., Ltd. (Dongguan, China). Chemically pure hydroxyl-terminated polydimethylsiloxane (OH-PDMS) was purchased from Jinan Xingfeilong Chemical Co., Ltd. (Jinan, China). Silane coupling agent (KH550), with a purity of 97%, was purchased from Shandong Yousuo Chemical Technology Co., Ltd. (Qingdao, China). Dimethyl silicone oil (100 cs) was purchased from Dow Corning Co., Ltd. (Auburn Hills, MI, USA). Silver-loaded titanium dioxide (TiO_2_-Ag, anatase, silver content 1%) was purchased from Zhejiang Zhitai Nano Micro New Material Co., Ltd. (Hangzhou, China). Deionized water was made in the laboratory.

### 2.2. Preparation of STASL and SLIPS

**Figure 1:**
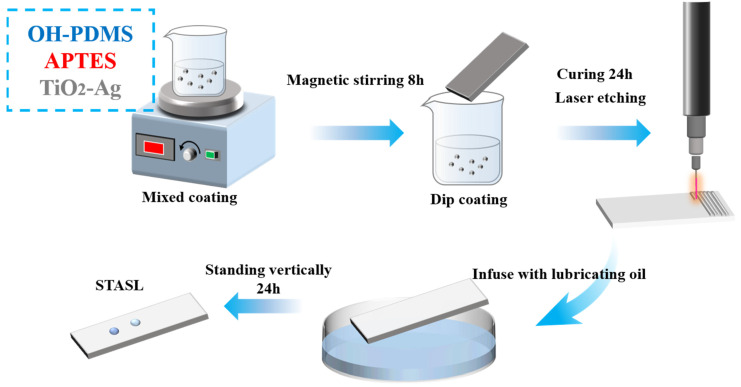
Preparation process of liquid super smooth surface STASL.

First, 10 g of hydroxyl, 5 g of silane coupling agent, and 0.2 g of TiO_2_-Ag particles were added to the beaker in sequence, and the particles were uniformly dispersed through continuous magnetic stirring for 12 h at room temperature and ultrasonic oscillation for 10 min. Concurrently, the titanium alloy plate was pre-treated by being ultrasonically cleaned with anhydrous ethanol and then dried to remove any dirt from its surface. After the reaction was completed, the pre-treated titanium alloy plate was immersed in the solution, slowly taken out, and allowed to dry at room temperature for 24 h to form a smooth surface of low surface energy with TiO_2_-Ag anti-bacterial granules (STA). Following this, a CO_2_ laser was used to etch the surface, creating a rough microstructure and forming a STA-based hydrophobic surface with rough structures (STAH). The focal spot scanning speed was 350 mm/s, the power was 6.0 W, and the line spacing was 250 μm. and uniform etching was performed in the X and Y directions. Finally, lubricating oil was poured onto the surface to prepare the STASL. The reaction flow chart is depicted in Figure 1.

The SLIPS was prepared on a titanium alloy plate using a fiber laser (SK-CX 30, Fiber laser, Shanghai, China). The focal spot scanning speed was 500 mm/s, the power was 15 W, and the line spacing was 100 μm. The uniform etching was performed in the X and Y directions to form a grid structure. Then, the samples were immersed in 1.0 wt% ethanol trimethoxy (1H,1H,2H,2H-perfluoro-n-octyl) silane solution for 30 min, and dried at 80 °C for 30 min. Finally, silicone oil was poured into the sample to obtain SLIPS [34].

### 2.3. Characterization

An optical contact angle measuring instrument (Solon, SL 200 KS, Shanghai, China) was used to measure the contact angle (CA) by dropping deionized water and silicone oil droplets onto the STAH surface at room temperature. The sliding angle (SA) was measured by water droplets to determine the sliding performance of the surface. The surface morphology was observed using field emission scanning electron microscopy (SEM, JSM-7900F, Japan Electronics Co., Ltd., Akishima, Japan), and the surface element composition was analyzed using energy dispersive spectroscopy (EDS). The 3D geometry of the surface was observed using a 3D surface optical profiler (ZYGO, New View 9000, ZYGO Corporation, Middlefield, CT, USA). The hardness of the STASL was measured by a QHQ-A pencil scratch hardness tester. Adhesion was measured by a SISI adhesive guide grid tester. Photos and videos of the surface were taken by a digital camera (Nikon, D5500, Tokyo, Japan).

### 2.4. Protein Adhesion Test

The adsorption experiment was carried out by using bovine serum albumin. Firstly, the sample was pretreated. For this, the sample was immersed in phosphate-buffer solution (0.1 M, pH = 7.0), washed and dried to remove surface dust, dirt and protein. Then, the standard protein solution was prepared and the standard protein curve at 280 nm wavelength was drawn.

The 25 mm × 50 mm × 2 mm sample was immersed in 30 mL standard protein solution and kept at room temperature for 12 h to achieve adsorption–desorption equilibrium. After adsorption, the samples were taken out, and the concentration of the remaining protein solution was measured using an ultraviolet-visible spectrophotometer (INESA Analytical Instrument, N4S, Shanghai, China) to calculate the amount of pollutants adsorbed on the membrane surface.

### 2.5. Bacterial Adhesion Test

Escherichia coli (*E. coli*), a common Gram-negative bacteria, was selected as the test strain for antibacterial test. Samples (30 mm × 60 mm), beaker and culture medium were first sterilized in a vertical pressure steam sterilizer at 121 °C for 1 h. The initial bacterial solution was diluted 6-fold to 10^−6^ times the original concentration. The sterilized sample was tilted at 25°, and then 10 mL of diluted bacterial solution was evenly sprayed onto the surface of the sample. After 2 h, the strain was transferred to the culture medium using the rubbing method, and cultured in a biological incubator at 37 °C for 24 h. The surface colonies were observed by plate counting.

### 2.6. Algae Cultivation and Settlement Test

In the anti-algae test, diatoms were selected as the test species. First, the beaker and transparent glassware were cleaned with deionized water and ethanol, and dried in ovens. The artificial seawater and algae nutrient solution were mixed in a ratio of 1000:1, and then the algae dilution was prepared according to the volume ratio of artificial seawater to algae liquid of 10:1. The mixed algae solution was incubated in a biological incubator at 25 °C for 48 h. To simulate the real marine environment, LED lights are used to provide 12 h of light, followed by 12 h of darkness. The bottle was gently shaken for 5 min every day to dissolve carbon dioxide and promote the growth and reproduction of algae. The samples were vertically immersed in the obtained algae solution and cultured in a biological incubator at 25 °C for 14 days. After incubation, the samples were taken from the algae solution, rinsed with distilled water and dried to observe the surface diatom adhesion of the samples.

## 3. Results and Discussion

### 3.1. Preparation and Surface Characterization of STASL Coating

**Figure 2:**
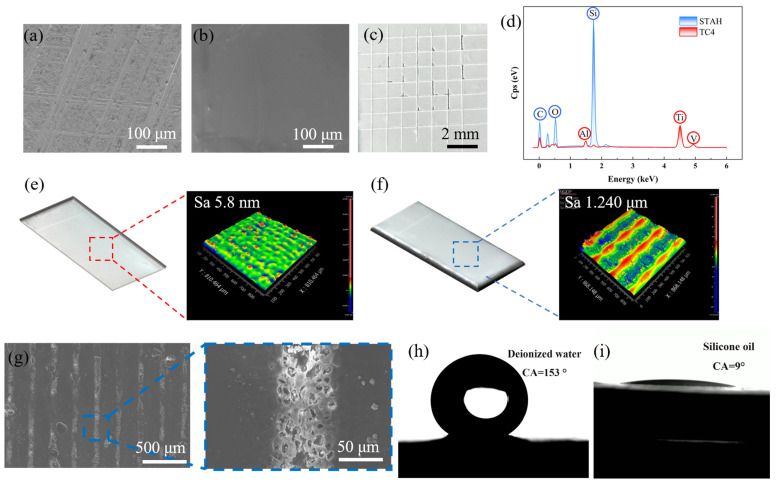
Surface characterization and composition. (**a**,**b**) Scanning electron microscopy images of untreated TC4 and STA surface; (**c**) surface adhesion test results of STA; (**d**) EDS spectra of STAH and TC4 surface; (**e**) optical images of STA surface and ZYGO images; (**f**) optical images of STAH surface and ZYGO images; (**g**) scanning electron microscopy images of STAH; (**h**) contact angle of pure water on STAH surface (153°), and (**i**) contact angle of silicone oil on STAH surface (9°).

Figure 2a,b show scanning electron microscope images of the untreated TC4 and STA surface. The TC4 surface displayed noticeable scratches and is quite uneven. In contrast, prior to laser etching, the STA surface was very smooth, with a surface roughness of only 5.793 nm (Figure 2e). Figure 2c presents the adhesion performance test results for the STA surface, which demonstrate good adhesion with a rating of 4B. The surface hardness was graded at 5B, reflecting the relatively low hardness of the silicone rubber material. Figure 2d displays the EDS spectrum of the laser-etched STAH surface compared to the untreated TC4 surface. The spectrum indicated that the STAH surface completely covers the TC4, with the laser etching not penetrating the STAH layer. The surface and ZYGO images following laser etching were presented in Figure 2f, revealing a uniform gully pattern, with surface roughness increasing to 1.240 μm. Scanning electron microscopy showed that the convex portions of the coating were ablated by the laser, resulting in numerous holes (Figure 2g) that facilitate lubricating oil storage. The contact angles of the laser-etched STAH surface were measured using deionized water and silicone oil. The contact angle for deionized water was found to be 153° (Figure 2h,i), while the contact angle for silicone oil was only 9°, indicating that the silicone oil spreads and penetrates the STAH surface rapidly. This demonstrated that the STAH surface had a better affinity for silicone oil than for deionized water. These favorable conditions collectively contributed to the formation of a slippery surface.

### 3.2. Slip Performance and Retention of Lubricating Oil

The retention of lubricating oil on the liquid slip surface is crucial, which directly affects the durability and service life of the coating. SLIPS was prepared and compared with the STASL. When the dip angle is 25°, the sliding velocities of different droplet volumes on the STASL and the TC4 surface were tested, and the sliding velocities of deionized water, HCl solution (pH = 1), NaOH solution (pH = 14), and NaCl solution (3.5 wt%) on the STASL were tested when the droplet volume is 25.4 μL. Rotating at 1000 rpm, 2000 rpm, 3000 rpm, 4000 rpm, 5000 rpm, 6000 rpm for 2 min respectively, the ratios of the loss mass of the lubricating oil after rotation to the initial adsorbed lubricating oil and the sliding angle SA were tested.

**Figure 3:**
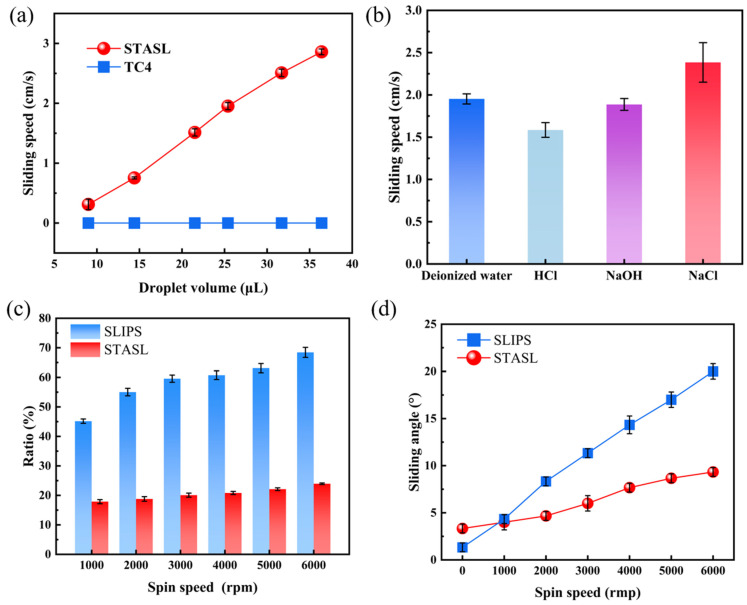
Lubricating oil retention and sliding performance of STASL: (**a**) sliding speed of different water droplet volumes; (**b**) sliding speed of four different test droplets; (**c**) ratio of sample mass loss to initial oil absorption at different rotation speeds, and (**d**) change in sliding angle with change in sliding speed.

Figure 3a and Table S1 show the sliding velocity of different water droplets. With the increase in the volume of the test water droplets, the sliding velocity of the water droplets on the STASL also showed a significant upward trend. When the volume of the water droplets was 36.4 μL, the sliding velocity of the water droplets was 2.859 cm/s. As shown in the Video S1, the droplets on the TC4 surface remained stationary as the volume of the droplets changed, which was not conducive to preventing marine biofouling.

**Figure 4:**
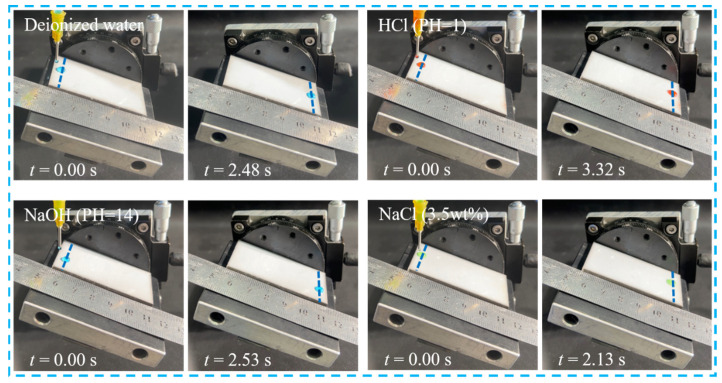
Sliding velocities of different droplet types on STASL.

Figure 3b, Table S2 and Figure 4 show the sliding speed of different droplet types. Deionized water, HCl solution (pH = 1), NaOH solution (pH = 14), and NaCl solution (3.5 wt%) all had a faster sliding speed on the STASL. The sliding speed of NaCl solution was the largest, which is 2.38 cm/s. This showed that the STASL can be applied to various liquid environments (as shown in Video S2).

Figure 3c and Table S3 are the ratio of the mass loss to the initial oil absorption of the sample at different spin speeds. At a lower speed, the mass loss rate of SLIPS was 45.17% after horizontal rotation at 1000 rpm, while the mass loss rate of the STASL was only 17.86% under the same conditions. With the increase in rotation speed, the mass loss rate of SLIPS showed an upward trend, while the mass loss rate of the STASL remained at about 20%. In the rotation of 6000 rpm, the mass loss rate of SLIPS reached 68.44%, which was 2.86 times that of the STASL (23.94%). Figure 3d and Table S4 show the sliding angle of the surface at different rotation speeds. The sliding angle of SLIPS was smaller than that of the STASL before rotation, and it had better sliding performance. However, after the start of rotation, the slip angle of SLIPS suddenly began to increase sharply, which was caused by the loss of lubricating oil at higher rotation speed. With the increase in rotation speed, the sliding angle of the STASL had a small increase. After rotating at 6000 rpm, the sliding angle was still about 10°.

### 3.3. Anti-Acid, Alkali and Salt Solution Fouling

**Figure 5:**
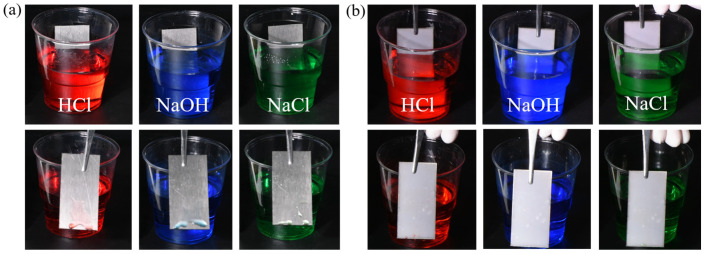
The residue of different solutions after soaking for 1 min: (**a**) TC4 surface, and (**b**) STASL.

The super-slippery STASL and untreated TC4 were immersed in HCl solution (pH = 1), NaOH solution (pH = 14), and NaCl solution (3.5 wt%), and then removed after 1 min. The residual solution on the STASL was examined, and the results are shown in Figure 5. As illustrated in Figure 5a, none of the three solutions remained on the STASL, while clear droplets were present on the TC4 surface. This indicated that the STASL exhibits superior resistance to fouling from acid, alkali, and salt solutions, demonstrating its suitability for environments with these conditions.

### 3.4. Silver Ion Leaching Test

**Figure 6:**
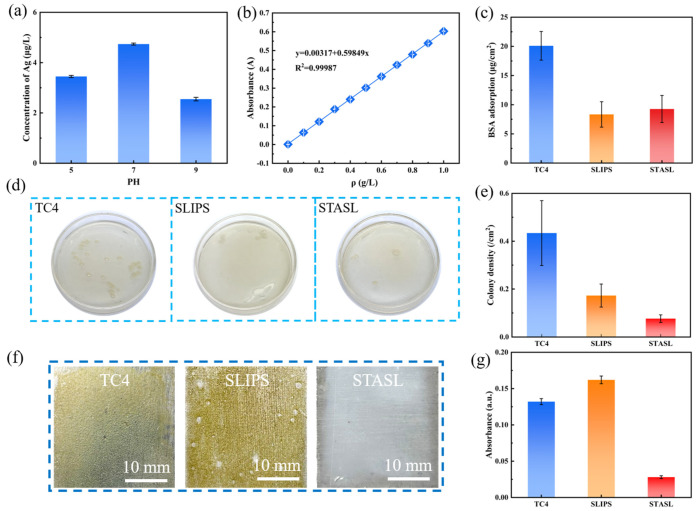
Antifouling performance of STASL. (**a**) Silver ion leaching concentration test results; (**b**) standard protein curve at 280 nm wavelength; (**c**) adhesion of different pieces of BSA adsorption; (**d**,**e**) adhesion of the sample to *E. coli*, and (**f**,**g**) adhesion of the sample to diatoms.

The super-slip STASL coating (20 × 20 mm^2^) after injection of lubricating oil was placed in a 100 mL solution with pH values of 5, 7 and 9. After 30 days, the content of silver in the solution was determined. The test results are shown in Figure 6a. It can be seen from the figure that silver ions were released into the solution in the coating at different pH values, and the silver content in the solution was the highest when the pH value was 7.

### 3.5. Marine Anti-Fouling Performance

The standard protein curve at 280 nm is presented in Figure 6b, alongside the BSA adsorption amounts for different surfaces shown in Figure 6c. Based on standard curve fitting, the BSA adsorption on the untreated TC4 titanium alloy surface was measured at 20.09 μg/cm^2^. In comparison, the SLIPS had an adsorption capacity of 8.34 μg/cm^2^, while the STASL showed a capacity of 9.24 μg/cm^2^, both showing good results in preventing protein adhesion. Notably, the STASL reduced BSA adsorption by 50.0% compared to untreated TC4, highlighting its effectiveness in preventing protein adhesion in the initial stages of marine pollution, primarily due to its super-slip properties that hinder protein adsorption.

Microorganisms, particularly bacteria like *E. coli*, are key contributors to biofilm formation during the second stage of marine biofouling. To investigate the antibacterial adhesion properties of the super-slip surface, *E. coli* was selected for testing. As illustrated in Figure 6d,e, a dense colony formed on the ordinary TC4 surface, with a density of approximately 0.43/cm^2^. In contrast, the SLIPS and STASL significantly reduced colony densities to about 0.17/cm^2^ and 0.08/cm^2^, respectively. Compared to untreated TC4, the STASL demonstrated a 77.8% reduction in *E. coli* adhesion. This indicated that both the SLIPS and STASL, containing nano-silver, effectively inhibited bacterial adhesion and growth. The synergistic effects of super-slip properties and antibacterial nano-silver in the STASL resulted in lower bacterial colony densities, preventing microbial attachment and subsequent fouling. In contrast, the AFSS prepared by Gu et al. [16], which combined capsaicin and sliding properties, had a colony density of 0.09/cm^2^ in the bacterial adhesion test. The STASL achieved the same anti-protein adhesion effect as AFSS, but AFSS contained fluoride, which is harmful to humans and the environment.

Algae are also crucial in the second stage of marine biofouling. Therefore, representative diatoms were tested for anti-adhesion properties across surfaces. The absorbance curve of diatoms (Figure S1) peaked at 440 nm, which was selected for subsequent UV–visible spectrometry to reflect diatom concentration. Untreated TC4, SLIPS, and STASL were cultured in diatom solutions for 14 days, and growth was assessed. Figure 6f,g show that diatom adhesion was significant on traditional SLIPS and untreated TC4, while the STASL exhibited almost no diatom adhesion. Compared with untreated TC4, the STASL reduced diatom adhesion by 78.8%, and compared with the SLIPS, it reduced diatom adhesion by 82.7%. The SLIPS prepared by Wang [40] was cultured in diatom solution for 14 days. Although the number of short navicular algae that adhered to the surface of the untreated titanium alloy was less than that of the untreated titanium alloy, the presence of diatoms can be clearly observed on the SLIPS. In static environments, the SLIPS showed limited anti-diatom effectiveness, but the presence of nano-silver in the STASL significantly contributed to its antibacterial properties, enhancing overall performance against fouling.

## 4. Conclusions

In summary, the laser etching of low-surface-energy surfaces formed by OH-PDMS, KH550 and TiO_2_-Ag nanoparticles and injected with silicone oil has enabled the development of an environmentally friendly super slippery surface (STASL) with favorable sliding properties and marine antifouling properties.

1. The results demonstrate that the STASL exhibits excellent lubricant retention and slip performance. With an increase in rotational speed, the mass loss rate of silicone oil in the STASL remains at approximately 20%, while the sliding angle remains at approximately 10°, indicative of an ultra-smooth surface.
2. In comparison to TC4, the STASL demonstrated superior sliding performance across a range of droplet types. At an inclination angle of 25° and a droplet volume of 25.4 μL, the sliding speed of a NaCl solution on the STASL was 2.38 cm/s, while at the same time, 36.4 μL of deionized water remained stationary on the TC4 surface.
3. Furthermore, the STASL demonstrated excellent anti-adhesion performance against acid and alkali salt solutions. The adhesion of both TC4 and STASL was simultaneously immersed in HCl solution (pH = 1), NaOH solution (pH = 14), and NaCl solution (3.5 wt%) for 1 min, after which the solutions were removed. The STASL exhibited minimal solution residue, whereas the TC4 surface displayed distinct droplets.
4. The STASL demonstrated excellent adsorption performance, reducing the adsorption rate of protein, bacteria and algae by 50.0%, 77.8% and 78.8%, respectively.
5. Furthermore, the STASL preparation process does not utilize any volatile organic reagents or toxic antimicrobial agents, which not only has a positive environmental impact but also ensures the safety of operators.

Consequently, we posit that this STASL can be employed as a promising antifouling coating for titanium alloys in marine engineering.

## Data Availability

Data are contained within the article and Supplementary Materials.

## Supplementary Materials

The following supporting information can be downloaded at https://www.mdpi.com/article/10.3390/ma17225598/s1: Figure S1: The absorbance curve of diatoms; Table S1: The values used to draw the sliding velocity images of different water droplet sizes (cm/s); Table S2: The values used to plot the sliding velocity images of different droplet types (cm/s); Table S3: The value of the ratio image of the mass loss of the sample to the initial oil absorption at different rotational speeds is drawn (%); Table S4: The value of the sliding angle image at different speeds is drawn (°); Video S1: Sliding speed of different water droplet volumes; Video S2: Sliding speed of four different test droplets.

## References

[1] Chang H., Wang X., Zhou L. (2014). Present situation and development trend of titanium alloy and its applications in ships. Materials.

[2] Jiang Q., Yi W., Yao L. (2016). The application of titanium and titanium alloys on foreign vessls. Ship Sci. Technol..

[3] Wang Y., Wang C.X., Li S.J., Li M.Z., Fu Z.R. (2019). Advances in marine biofouling control. Neijiang Technol..

[4] Shang T.L., Wang L., Shang Y.C. (2002). Marine Organisms Attached to Ship Bottom and Their Control. Mar. Technol..

[5] Kalmuck K.M. (1997). Development of a dynaJet cavitating water Jet cleaning tool for underwater marine fouling removal. Am. Waterjet Conf..

[6] Kawabe A. (2004). Development of antifouling technologies for heat exchanger. Sess. Org..

[7] Soren H. (2002). Seawater soluble pigments and their potential use in selfpolishing antifouling paints: Simulation-based Screening tool. Prog. Org. Coat..

[8] Legg M., Yücel M.K., Kappatos V., Selcuk C., Gan T. (2015). Increased range of ultrasonic guided wave testing of overhead transmission line cables using dispersion compensation. Ultrasonics.

[9] Legg M., Yücel M.K., Garcia De Carellan I., Kappatos V., Selcuk C., Gan T.H. (2015). Acoustic methods for biofouling control: A review. Ocean. Eng..

[10] Xu Z., Ouyang Q., Yi D. (2012). Overview and development trend of marine fouling biological control methods. Corros. Sci. Prot. Technol..

[11] Liang C., Gu Q., Wu Q. (1997). Antifouling treatment technology of electrolytic seawater. East China Sea.

[12] Lv Z.M. (2002). The prevention and control technology of fouling organisms in mariculture nets. Chin. Aquat. Prod..

[13] Liu S.S., Yan T. (2006). Status and Prospects of Marine Biofouling Control. Oceanogr. Res..

[14] Wake H., Takahashi H., Takimoto T., Takayanagi H., Ozawa K., Kadoi H., Okochi M., Matsunaga T. (2006). Development of an electrochemical antifouling system for seawater cooling pipelines of power plants using titanium. Biotechnol. Bioeng..

[15] Xie Q., Pan J., Ma C., Zhang G. (2019). Dynamic surface antifouling: Mechanism and systems. Soft Matter.

[16] Liu X., Gu X., Zhou Y., Pan W., Liu J., Song J. (2023). Antifouling Slippery Surface against Marine Biofouling. Langmuir.

[17] Silkinaa B.A., Bazes A., Mouget J.L., Bourgougnon N. (2012). Comparative efficiency of macroalgal extracts and booster biocides as antifouling agents to control growth of three diatom species. Mar. Pollut. Bull..

[18] Almeida J.R., Vasconcelos V. (2015). Natural antifouling compounds: Effectiveness in preventing invertebrate settlement and adhesion. Biotechnol. Adv..

[19] Zhang X., Brodus D., Hollimon V., Hu H. (2017). A brief review of recent developments in the designs that prevent bio-fouling on silicon and silicon-based materials. Chem. Cent. J..

[20] Nir S., Reches M. (2016). Bio-inspired antifouling approaches: The quest towards non-toxic and non-biocidal materials. Curr. Opin. Biotechnol..

[21] Ware C.S., Smith-Palmer T., Peppou-Chapman S., Scarratt L.R., Humphries E.M., Balzer D., Neto C. (2018). Marine Antifouling Behavior of Lubricant-Infused Nano wrinkled Polymeric Surfaces. ACS Appl. Mater. Interfaces.

[22] Burgess J.G. (2003). The development of marine nature product based antifouling paint. Biofouling.

[23] Gao X., Zhou J., Du R., Xie Z., Deng S., Liu R., Liu Z., Zhang J. (2016). Robust Superhydrophobic Foam: A Graphdiyne-Based Hierarchical Architecture for Oil/Water Separation. Adv. Mater..

[24] Yan D., Lu Y., Lin J., Li W., Song J. (2024). Enhancing water transportation capacity by asymmetrical patterned surface with super-wettability. Appl. Phys. Lett..

[25] Yan D., Lin J., Zhang B., Zhang S., Ling S., Song J. (2024). Drag reduction and antifouling of a spontaneous fast moving air film. J. Mater. Chem. A.

[26] Zhang J., Chen Y., Zhang Y., Wu S., Sun J., Liu X., Song J. (2024). Fabrication and Energy Collection of Superhydrophobic Ultrastretchable Film. Adv. Funct. Mater..

[27] Bers A.V., Díaz E.R., Da Gama B.A.P., Vieira-Silva F., Dobretsov S., Valdivia N., Thiel M., Scardino A.J., McQuaid C.D., Sudgen H.E. (2010). Relevance of mytilid shell microtopographies for fouling defence—A global comparison. Biofouling.

[28] Nhung Nguyen T.P., Brunet P., Coffinier Y., Boukherroub R. (2010). Quantitative Testing of Robustness on Superomniphobic Surfaces by Drop Impact. Langmuir.

[29] Zhang S., Liang X., Teng X., Gadd G.M., McGrath J.W., McCoy C.P., Zhao Q. (2023). Enhanced anti-biofilm and anti-protein adsorption properties of liquid-infused silver-polytetrafluoroethylene coatings. Appl. Surf. Sci..

[30] Epstein A.K., Wong T.-S., Belisle R.A., Boggs E.M., Aizenberg J. (2012). Liquid-infused structured surfaces with exceptional anti-biofouling performance. Proc. Natl. Acad. Sci. USA.

[31] Ouyang Y., Zhao J., Qiu R., Hu S., Niu H., Zhang Y., Chen M. (2020). Nanowall enclosed architecture infused by lubricant: A bio-inspired strategy for inhibiting bio-adhesion and bio-corrosion on stainless steel. Surf. Coat. Technol..

[32] Li F., Hu Y., Feng X., Tian G. (2024). Environmentally friendly SLIPS coating based on flexible sponge: A novel approach to antifouling for ships. Colloids Surf. A Physicochem. Eng. Asp..

[33] Xiang T., Chen X., Guo Z., Wang J., Cui L., Qiang Y., Zhang S. (2024). Robust solid slippery surface for anti-corrosion: Experimental and simulation. Prog. Org. Coat..

[34] Ma J., Pan W., Li Y., Song J. (2022). Slippery coating without loss of lubricant. Chem. Eng. J..

[35] Fan H., Guo Z. (2021). WO_3_-based slippery coatings with long-term stability for efficient fog harvesting. J. Colloid Interface Sci..

[36] Prieto-López L.O., Herbeck-Engel P., Yang L., Wu Q., Li J., Cui J. (2020). When Ultimate Adhesive Mechanism Meets Ultimate Anti-Fouling Surfaces-Polydopamine Versus SLIPS: Which One Prevails?. Adv. Mater. Interfaces.

[37] Feng L., Jiang H., Luo H., Ge F., Qiu Y. (2024). Research progress of nanometer Ag-TiO_2_ composite antimicrobial materials. Railw. Energy Conserv. Environ. Prot. Saf. Health.

[38] Ren L.T., Xu Y.Y., Ye Z.X., Ren G., Yin L.S. (2023). Antimicrobial principle of nanosilver and research progress in antimicrobial coatings. Mater. Her..

[39] Janík R., Kohutiar M., Dubec A., Eckert M., Moricová K., Pajtášová M., Ondrušová D., Krbata M. (2022). DMA Analysis of Plasma Modified PVC Films and the Nature of Initiated Surface Changes. Materials.

[40] Wang Y.J. (2019). Preparation of Bionic Smooth Titanium Alloy Surface and Its Antifouling and Corrosion Resistance Behavior.

