# Supplementary material for: Antifouling Slippery Surface with Enhanced Stability for Marine Applications
Source: Materials (Basel). 2024 Nov 15;17(22):5598. doi: 10.3390/ma17225598 (PMC11595577; doi:10.3390/ma17225598)
Supplement: Supplementary file 1 [file materials-17-05598-s001.zip › materials-3296267-Supplementary Materials.pdf]

High-performance Super-slips Surfaces for Anti-biological Corrosion

Yun Li [1], Yuyang Zhou [1], Junyi Lin [1], Hao Liu [1], Xin Liu [1,*];

[1] State Key Laboratory of High-Performance Precision Manufacturing, Dalian University of Technology,
Dalian 116024, China; hebly@mail.dlut.edu.cn（Y. L.）; zyuyang12@163.com (Y.Z.); linjy1372@163.com (J.L.); 15141623006@163.com （H. L.）;
[*] Correspondence: xinliu@dlut.edu.cn

Figures

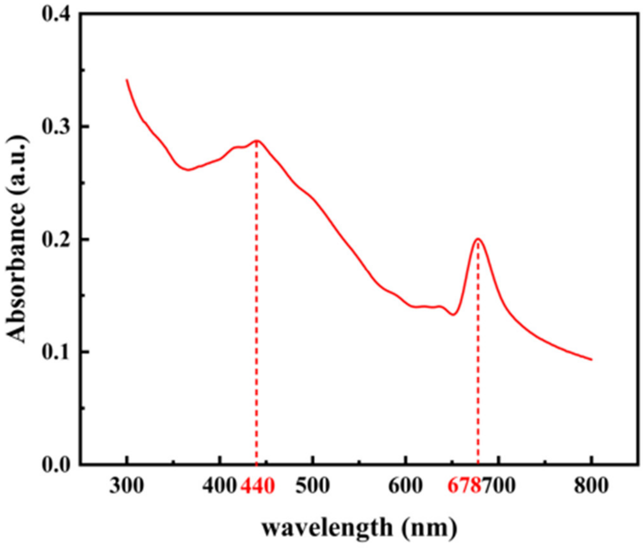

Figure S1. The absorbance curve of diatoms.

Tables

Table S1. The values used to draw the sliding velocity images of different water droplet sizes. (cm/s)

Surfaces \ Volume/μL	9	14.4	21.5	25.4	31.7	36.4
STASL	0.31	0.75	1.51	1.95	2.51	2.86
TC4	0	0	0	0	0	0

Table S2. The values used to plot the sliding velocity images of different droplet types. (cm/s)

Surfaces \ Droplet type	Deionized water	HCl (pH=1)	NaOH (pH=14)	NaCl (3.5 wt%)
STASL	1.95	1.59	1.89	2.38

Table S3. The value of the ratio image of the mass loss of the sample to the initial oil absorption at different rotational speeds is drawn. (%)

Spin speed /rpm Surfaces	1000	2000	3000	4000	5000	6000
STASL	17.86	18.78	20.07	20.81	22.10	23.94
SLIPS	45.17	55.03	59.57	60.75	63.12	68.44

Table S4. The value of the sliding angle image at different speeds is drawn. (°)

Spin speed /rpm Surfaces	0	1000	2000	3000	4000	5000	6000
STASL	3.3	4.0	4.7	6.0	7.7	8.7	9.3
SLIPS	1.3	4.3	8.3	11.3	14.3	17.0	20.0

Videos

Video S1: Slip velocity of droplets of different sizes on the STASL surface.

Video S2: Slip velocity of different types of solutions on the STASL surface.
